# Ghosts, Patent and Prescriptive

**Published:** 1863-07

**Authors:** 


MEDICAL CRITIC

AND

PSYCHOLOGICAL

JOURNAL.

JULY, 1863.

### Art. I.—GHOSTS, PATENT AND PRESCRIPTIVE.

"Je n'invente pas, je raconte."—Cousin.

Our ghost-seers would do well to profit by the larger experience of some of their Eastern brethren. In dealing with the hyperphysical as the physical, it is always advisable to proceed from that which is best known to that which is least known. This is more particularly the case when, as in recent so-called "spirit manifestations," we are called upon largely to modify or entirely to set aside time-honoured convictions of the supernatural. Now, Persia and conterminous countries have luxuriated in an affluence of spirits from time immemorial. There the true believer is never out of ear-shot of denizens of the other world, and he is apt to be elbowed by them at any moment in the highways and byeways, or in places of public resort. The ancient rabbins, who at times were subjected to a ghostly pressure so great that their apparel might be seen to fret beneath the spectral attrition, did not move amidst a denser mob of spirits than the true believer of our own day. He can never be certain, from an ordinary examination, such as we are accustomed to bestow upon strangers, that the unknown whom he may accidentally jostle in the streets, or who may sit beside him in the coffee-shop, or prostrate themselves with him in devotion in the mosque, may not be spirits. The inability to distinguish a spectral from a real personage at a glance, when the former choose to frequent the resorts of men under the ordinary aspects of true believers, would signify little, if it were not that the spirits are prone to take offence and swift to inflict punishment when offended. In fact, they are a vindictive race, the good spirits equally with the evil; the evil, by reason of their ill-begotten nature; the good, consistently with their religious professions. For the good spirits are true Mohammedans, and as such they are permitted by the Koran to take vengeance at least "equal to the injury that hath been done them."—(c. xxii.)

The religious and somewhat pugnacious character of the higher orders of Oriental spirits is not an isolated phenomenon. It is common, perhaps, to the popular conceptions of the spirit-world of all races and times. The modern spirits of whom Mr. Howitt is now constituted the great historian, and Mr. D. D. Home is one of the chiefest apostles, possess this character in a very appreciable degree. They pride themselves on their *quasi* Christianity, and on the fact that the present civil war in America was mainly brought about by their machinations. "The Northern people as a body," said the spirit of Washington, in a communication made from the spheres on the 7th of May, 1861, "have long looked upon slavery with disgust. They have felt that it was the great blot upon their name as a nation. But they had not, and never would have had, the power to relieve themselves from its crushing weight, had they not been aided and abetted by spirits who stirred up the elements of discord and strife between them and their Northern brethren. The South, waxing haughty and presumptuous by long immunity in wrong, and fancying in the arrogance of feeling engendered partly by her own dominant state which slavery has developed, and partly by the lying fabrications of newspaper editors, who, if they did not wilfully lie, perverted the truth to such an extent that the Southern people were led to believe, in their simplicity, that the great majority of the minds of the North were on their side—the South, I say, instigated by these things, and still more worked upon by the unseen work of spirits, their proud and defiant natures naturally attract around them, have been hurried, blindly and impetuously, into this war—a war fatal in all worldly points of view to them, but rich in blessings to this and all succeeding times, morally and spiritually."* This is as pretty a genealogy

* *Essays on Various Subjects; intended to Elucidate the Causes of the Changes coming upon all the Earth at this present Time, and the Nature of the Calamities that are so rapidly approaching, &c. &c.* By Joshua, Cuvier, Franklin [Thomas Paine, Jesus the Christ], &c. Given through a Lady. New York, 1861. p. 194. "The Essays in this book," says the spirit either of George Fox, or of JOHN the Apostle, it is not very clear which, in the Preface, "have been, each of them, the united efforts of several spirits, one taking up one part of the theme, another spirit another part, when he could bring the ideas with greater facility to the medium. *She has no proof of this, excepting in the changes of handwriting which occurred as the different influences took control.* The spirits, however, who have most generally written have been Joshua, Cuvier, Franklin, Fox, Luther, and the Apostle John. Just as the nature of subject was more nearly in rapport with a particular spirit's magnetism, was that spirit drawn to give the influence."

as could be desired of the most deplorable civil strife which the world has as yet seen. Was it, indeed, the spirit of Washington who made the communication,* or the spirit of that candidate for the Presidency who, as the inimitable Mr. Ezekiel Biglow records, wrote in answer to certain questions propounded to him :—

> " Not but wut abstract war is horrid,
> I sign to that with all my heart,—
> But civlyzation *doos* get forrid
> Sometimes upon a powder cart."

Knowing the revengeful character of the spirits who may be about him, visibly or invisibly, it is necessary for the true believer to be constantly on his guard, and experience has taught him one or two precautions which suffice for all ordinary occasions. For the same reason that the Irishman terms the fairies " Good people," the true believer, when speaking of spirits, terms them " Better ones than ourselves ;" and the latter, if a wise individual, is careful never to cast anything away, particularly anything that is filthy or objectionable, without exclaiming, " By your leave !" The spirits are singularly irate if aught is thrown upon them which defiles or injures them, or if they are spoken of in any but complimentary terms. The story of the Merchant and the Genius in the Arabian Nights is an apt illustration. The unfortunate traveller had cast away a date stone carelessly. The stone struck a young genius upon the chest and killed him. Immediately an enormous Afrite, the father of the slain genius, appeared to take summary vengeance. Few will have forgotten in what manner the merchant escaped death. Let it not be supposed that such an event as this was peculiar to remote times. Scarcely a lustrum and a half ago, we saw a fine young Turkish sailor struck down suddenly with convulsions. " What has occasioned this ?" we asked of an attendant. " Ah, Effendim," he replied, " Omar cast some foul water through the doorway, yesternight, without exclaiming ' Destur !' (By your leave). The water has befouled a Jinn, and he has punished the offender. Omar will die ; help is unavailing for him." In twelve hours, the poor fellow was dead, and his companions took the lesson to heart. Happily the Moslem spirits are as amenable to courtesy as prone to anger ; and the simple utterance of the phrase (so

---

* In another portion of the communication, Washington is made to say of the North—" Your armies will be victorious in the South ; but shall you rejoice in victory ?" The answer is in the negative ; and the North is told that it will mourn in anguish of spirit over the desolation it has caused in the South, and over that love of gold which had determined the fearful troubles now or about to be experienced.

vulgarized at our railway stations), "By your leave, gentlemen!" is all that is needed to prevent unhappy contingencies when the spirits are invisible.

In intercourse with our modern spirits it is also found that courtesy is very requisite. The expression of impertinent doubts, or the slightest neglect of those polite observances which society demands in ordinary life, is apt to be resented. Fortunately, the resentment is usually confined to the withdrawal of all communications with the offending individual. This, however, is to be attributed rather to forbearance than want of power. A Dr. Gridley records an instance, by no means an isolated one, we believe, in which the "most wrangling and horrible convulsions" were caused by spiritual agency.

The chief difficulty with the Eastern spirits arises, however, when they assume the aspect and garb of common humanity, and so transformed frequent public places. The thought is not pleasant of being hail-fellow-well-met with a spirit without knowing it. Not only is the true believer exposed to the chance of committing some egregious solecism, spiritually considered, but usually there is a spice of malice in a spirit when appearing in human form, which is liable to be brought into play without any sufficient cause being apparent. It must be understood that when spirits thus mix with men, in the guise of men, unless the trick of detection is known, there is no distinguishing them from ordinary human beings. Their corporeity is not a whit less substantial than that of commonplace humanity, their clothing as tangible as that fabricated by human hands. This high degree of reality, or rather materiality, of the Eastern spirits is peculiarly instructive. The remarkable physical qualities of modern Occidental spirits, when they have manifested themselves otherwise than indirectly, have been perpetual stumbling-blocks to the sceptical. It was difficult to conceive the notion of an incorporeal being indistinguishable from a corporeal: yet the conception was necessary if the supra-mundane character of certain "spirit manifestations" was to be admitted. For example, in an account of a sitting with Mr. Home, originally published in the *Spiritual Magazine*, and now republished in that gentleman's autobiography,* we read: "As soon as this (spectral performance on the accordion, with a drumming accompaniment of knocks) ceased, the table rose up in the air, and floated away from us high above our heads, passing over sofas and chairs in its way. We were naturally greatly interested at this wonderful manifestation, and followed it into the darker part of the room; and here arose a scene of indescribable con-

* *Incidents of my Life.* By D. D. Home. London, 1863. p. 148.

fusion, but still producing feelings in no way unpleasant, though we knew not, when we touched each other, who were spirits and who were human beings." Again, Mr. Home tells us that in one of his sittings, "there came a hand on his head and forehead as *much like flesh and blood* as any he ever felt, *only somewhat cold*."—(p. 51.) Of another hand in another sitting, he says, "The hand afterwards came and *shook hands* with each one present: I felt it minutely. It was tolerably well and symmetrically made, though not perfect; and it was *soft* and slightly *warm*. It ended at the wrist."—(p. 61.) Dr. J. I. G. Wilkinson writes, of one of Mr. Home's sittings, "I put my hand down . . . . . and was regularly stroked on the back of it by a soft palpable hand as before . . . . . During this *séance* I had the border of a white cambric handkerchief just appearing out of the side pocket of my paletot, which was open; and though I could see no agency, I felt something twitching at the handkerchief, and very gradually drawing it from my pocket. Simultaneously with this, my eldest daughter, who sat opposite to me, exclaimed, 'Oh! I see phosphoric fingers at papa's pocket!' and now, visibly to all, the handkerchief was slowly pulled out, and drawn under the table; whilst, at the same time, I felt an arm that was doing it, but which was invisible to me. At this time I was at least three feet from Mr. Home, with a person between us, and he was absolutely passive. The feeling I had was of nudges, as distinct as ever I felt from mortal limb, and that on my breast and arm, which were above the table; and yet, though the operation of abstracting my handkerchief was going on visibly to all, the rest of the circle, as well as myself (all except my eldest daughter), could see nothing. I can swear that there was no machinery, unless the *skin, bone, muscle, and tendons of an unseen hand, forearm, and elbow*, deserve the name."—(p. 76-7.) The same gentleman writes of another sitting, and of another hand, "the palm was laid flat upon my brow, where it remained for several seconds. It was warm and human, and *made of no material but softest flesh*."—(p. 82.)

It needs but a brief acquaintance with Oriental spirits to learn that the remarkable physical characteristics of the supposed spectral phenomena thus described, are quite consistent with known and recognised forms of belief in the supernatural. Nay more, Mr. Howitt has learnedly and ably shown, in his recently published History of the Supernatural,* that if the too narrow con-

* *The History of the Supernatural in all Ages and Nations, and in all Churches, Christian and Pagan: demonstrating a Universal Faith.* By William Howitt. 2 vols. London, 1863.

ception commonly entertained in this country, and among many Christian nations, of the physical capabilities of spiritual agents, be extended so as to include the conceptions of all nations in all ages, and of all churches, Christian and pagan, the difficulty of admitting the supra-mundane origin of the "spirit manifestations," in their entirety, at once vanishes.

The chief apologists of the present "spirit" movement are, indeed, curiously anxious to have it understood that the "spirit-manifestations" of our own day are by no means novel, but that they have been peculiar to supernatural interferences in mundane affairs from very remote times. They ignore the fact, or regard it as of slight import, that an argument had been based on this peculiarity, to the effect that the later manifestations were but a re-awakening of certain delusions, and delusive notions, to which man was, and had been, at all times liable. It is not very clearly explained by the apologists, how it comes to pass that the further we recede from our later civilization, the grosser are the popular conceptions of spirit. As, however, modern spirits tell us that in proportion as scepticism decreases, so their capability of material projection will increase; it may be useful to interpolate a few illustrations of the degree of projection recorded in earlier times. In a curious and more ancient version than that commonly known of the story of *Ali Baba and the Forty Thieves*—a version which we learned from a native of Erzeroum—the thieves are represented by a band of evil spirits (spoken of under the generic term of Peris), and they are got rid of by cutting the throat—a lethal process much more consistent with the goat-skin oil bags in which they were concealed, than scalding by boiling oil.

Putting genii to death with the knife, as related in this story, is true to the spirit of ancient mythology and middle-age romance, in which the vague spectral apparitions of Western ghost-lore in modern times have a very subsidiary position. It is true, also, to the thorough-paced materiality of spirits in the East at the present time, as has already been seen. Before Troy Diomede pierced the hand of Venus with his spear, and she screamed aloud while immortal blood flowed from the wound, "ichor, such, to wit, as flows from the blessed gods;" the same hero pierced Mars in the flank, "and tore his beautiful skin," while the brazen god "roared as loud as nine or ten thousand men roar in war, joining in the strife of battle." When England, according to Procopius, was a depôt, or occidental Hades, for the ghosts of the Continent, the fishermen, whose duty it was to convey them to the island, would notice that their boats sunk to the gunwales with the *weight* of the spectral cargo; the spectres in the Anglo-Saxon poem of

*Beowulf* are substantial, destructive beings; and the Nibelungs, and dwarf Albric, of the *Nibelungenlied,* have no signs of their supernatural character about them, save their surpassing physical powers. The fairies of the middle ages were admirable compounds of spiritual and material properties. Their knights were excellent, well-limbed fighting men, who fought with the sword, and who were killed by the sword; and the fairy ladies were as tangible as any one could desire them to be—a fact which Mr. A. Hughes has admirably rendered in his painting "La Belle Dame sans merci," recently exhibited at the Cosmopolitan Club. The spirit which thrust aside Sacripant and Rinaldo, when contending about Angelica, had no signs of incorporeality about it.* When Duke Robert of Normandy was assailed by a spirit, while praying at night in an abbey, he cleft the intrusive spectre in twain with his two-handed sword, and so ended it. The Deevs, as the wonderful prowess of the mighty Tahmuras, and the mightier Rustem, showed, are subject to death from the sharp-cutting steel; and the Genii fall beneath it. So that to put upwards of forty spirits to death by ghastly incision, is no impudent stretch of the imagination, but an orthodox and feasible mode of getting rid of such intruders, according to ancient rules of spectral corporeity.

The lesson is not without practical value. For now that spectres of a nature so corporeal that they cannot be distinguished by the touch from ordinary beings, spectres possessed of thews and sinews, are moving freely among us; although we have set aside the more vehement means of getting rid of obnoxious characters practised by our forbears, there can be no good reason why, if troublesome, they should not be given over to condign punishment, with the aid of that grand moral resource of the 19th century, when offenders are in question—the policeman.

A further examination of the characteristics of Oriental genii throws light upon other and equally extraordinary physical phenomena with those already mentioned, exhibited during "spirit-manifestations." Few things have excited greater doubt than the asserted elevation of heavy bodies (as in an instance already cited) or human beings in the air, without perceptible cause. Mr. Home's aërial flights are notorious. Assuming, for the moment, the correctness of the facts averred, a doubt might still reasonably rest upon the conclusion that the phenomenon was occasioned by spiritual agency. Now, no fact is better established in the whole range of Oriental spirit-manifestations,

* *Orl. Fur.*, B. ii.

than that a like phenomenon is frequently observed, and this under circumstances less open to question than in the occidental manifestations. It might be sufficient, and Mr. Howitt would no doubt justify and approve of the reference, to point out the numerous phenomena of the character referred to, recorded in the *Arabian Nights*, as, for example, in the stories of *Aladdin and the Wonderful Lamp*, *Prince Camaralzaman and the Princess Badoura*, and *Prince Achmet and the Peri Princess Banou.* It will be more satisfactory, however, and more interesting to supply an illustration of recent date. The following is from our own knowledge, and is peculiarly worthy of note as occurring at a time when Mr. Home's "levitation" (as he phrases the phenomenon of elevation) was exciting the wonder of this country and America. Late one evening, during the autumn of 1855, a very bulky Ottoman naval officer, who had been ashore dining with a friend, was making his way along the narrow streets of Sinope towards the beach, when suddenly a gigantic Genii stood before him. A thick veil of long grey hair, through which the spirit's eyes could be seen glowing balefully, hid the face and fell over the shoulders. Seizing the terror-stricken officer by the waist, the Genii, without uttering a word, hurled him high above the roofs of the houses into the air. Again and again was the unfortunate victim tossed up, as if he had been a child's ball; then the spirit, placing him roughly on his feet, with a malignant laugh disappeared. There could not well be an error about this occurrence. It happened on a bright and clear moonlight night, when objects in the streets could be seen almost as clearly as in daylight, and not, as in Mr. Home's aërial experiences, in the dark, or, at the best, a doubtful twilight. An attendant was with the officer, and witnessed the spectral assault. But it was only necessary to hear the victim's own version of the story, on the morning after the rencontre, to be convinced that he was telling what at least he believed to be the sober truth. But this need not be insisted upon. Mr. Howitt has so largely argued the truthfulness of cognate stories, and the beliefs out of which they arise, that the probability, if not certainty, of this story may be admitted without hesitation. It was said at the time (by a Christian, however), that the officer was drunk when he left his friend's house. There is some doubt of this. He was a Mussulman of the old school, although he had travelled, having spent several months in England. But admitting that, after the fashion of many Turks who have visited the West and picked up a few Western vices, he occasionally tippled, it is not to be forgotten that Jung Stilling, a "Spiritualist" (to use the current slang of the Mediumistic faith) of no ordinary calibre, was inclined to maintain that spiritual perception may be promoted by drinking ardent spirits.*

It is not necessary to examine further in detail the points of resemblance existing between the "spirit manifestations," and contemporary forms of spirit belief. Those who would pursue the subject will find abundant material for the purpose in Mr. Howitt's history. Sufficient has been said to show that the extraordinary physical exercitations and qualities of recent presumed spirits in this country and America, and certain psychical peculiarities, are not isolated phenomena, although incongruous with the notions of spirit and spiritual influence commonly received in civilized Christendom. But there is a question deserving of attention which may perhaps best be considered in this place, as much light may be thrown upon it, derived from one of the least familiarly known and most barbarous of the Mohammedan nations of Africa. The modern spirits, as becomes the epoch of their development, have shown from time to time utilitarian tendencies. One of the spirits holding communication with Dr. Hare, a famous scientific American medium, once made itself very useful (the story has a place in Mr. Howitt's history), by transacting some money business for him a hundred miles away. It is obvious that, if the spirits are about to give us aid after this fashion, they will much more readily convince the world of the advantageousness and reality of their present manifestations, than by attempting the indoctrination of transcendental teachings, which are unfortunately too apt to induce a doubt of their supramundane origin. Now, the experience of the African nation referred to may be profitably commended to the attention of ghost-seers. It is to be hoped that Messrs. Speke and Grant, now on their return from the head waters of the Nile, may bring us additional information on this subject. And here a remonstrance may be justly addressed to our spirit mediums. Why did they suffer us to remain in doubt of the safety, and at length permit us to believe the death of Mr. Petherick? Why did they keep us in the dark respecting the course of Messrs. Speke and Grant? Surely the spirits of some of the geographers, naturalists, and other scientific men, who have of late been so busy in conveying to us descriptions (including approximative measurements) of the spheres, and teaching us new and improved systems of philosophy and morals, and who regret so much the as yet imperfect communication between the spirit-world and man; surely these spirits might have taken a little interest in

* *Theory of Pneumatology.* Jackson's Translation. London, 1834. p. 182.

the great geographical puzzle which Messrs. Speke and Grant were engaged in solving; more especially as in the districts traversed by the Upper Nile they would have learned that the facility of corporeal communication they so much desired here, had long been enjoyed by the spirits of those less favoured and more barbarous regions? Few will have forgotten the story of the bottled Afrite in the *Arabian Nights.* It is an error to look upon this bottling as a sheer freak of the fancy, such as Le Sage's description of Asmodeus corked up in a phial in the astrologer's garret. In Darfur at the termination of last century (and doubtless the practice exists still), the natives were accustomed to bottle up spirits for domestic use—not ardent spirits, we do not seek to pun—as regularly as we bottle our cognac, or whisky, or gin. The Arab merchant, Sheikh Mohammed of Tunis, whose relation of a journey to two of the most remarkable black kingdoms of Central Africa, Darfur and Wadaï, was made known to us by Dr. Perron's translation, of which Bayle St. John published an abridgment in English, tells us that the most singular thing he heard whilst in the mountains of Darfur was this, that the genii act as guardians of the cattle. "It is for this reason," he writes (we use the words of the abridgment), "that the herds are left to wander where they will. Many persons assured me seriously that if any one passing near a flock, and seeing it without a guard, should attempt to steal a sheep or cow, and to kill the beast, his hand, still armed with the knife, would remain fastened to the throat until the arrival of the owner. I have a hundred times heard instances of protection afforded by the genii, but was at first inclined to reject the whole as lies and dreams. But this is what happened to myself. Being near the Marrah Mountains, I went to a person of Numlah, to question him about genii. On drawing near his hut I saw nobody, but began to call him by his name. Then a loud and terrible voice, which made me shiver, shouted, 'Akibe!' that is to say, 'He is not here.' Nevertheless, I was going to advance and pursue my inquiries, when an individual, who was passing by me, took me by the arm and drew me away, saying, 'Be off! be off! He who speaks to thee is not a human being.' 'And what is he, then?' 'He is the guardian genius of the hut. Nearly every one of us is thus protected. We call the genii in Forian, Damzog!' Upon this I feared and withdrew. On returning from the Marrah to the Fasher, I went to visit the Shereef Ahmed Bedawee, who had brought me from Cairo to Darfur, and related to him this adventure and my terror. 'The man was perfectly right,' said Ahmed, who went on to relate to me things still more wonderful. 'At the time when I first began to trade, my friend, I often heard that damzogs could be bought and sold, and that to procure one I must apply to the owner of a damzog, and discuss the price with him. When the bargain is concluded, it is necessary to give a large gourd of milk to the seller, who takes it to his house, where are his damzogs. On entering he salutes them, and goes and hangs up his vase to a hook, saying, 'One of my friends—such a one—very rich, is in fear of robbers, and asked me to supply him with a guardian, will one of you go and live in his house? There is plenty of milk there, for it is a house of blessing, and the proof thereof is, that I bring you this kara of milk.' The damzogs at first refuse to comply with the invitation. 'No, no,' say they, 'not one of us will go.' The master of the hut conjures them to comply with his desires, saying, 'Oh! let the one that is willing descend into the kara.' He then retires a little, and presently one of the damzogs is heard to flop into the milk, upon which he hastens and claps upon the vase a cover made of date leaves. Thus stopped up he unhooks the kara, and hands it over to the buyer, who takes it away and hangs it on the wall of his hut, and confides it to the care of a slave or of a wife, who every morning comes and takes it, emptying out the milk, washing it and replenishing it, and hanging it up again. From that time forward the home is safe from theft or loss. For my part, I believed all these things to be absurdities. Well, my wealth increased; but my slaves and servants constantly robbed me. Vainly did I have recourse to all kinds of means to prevent them; I was always duped. One day I complained to a friend, who recommended me to buy a damzog, certifying that I should be thus effectually protected. The desire of preserving my property induced me to comply, and so I went to a possessor of damzogs, and bought one in the way I have described. I appointed a slave to watch over the kara, and from that day forward I was free from care. I even left my warehouse door open, and nobody in my absence dared to approach . . . . It has often been related to me, that among the great drums or tymbals, preserved in the dwelling of the Sultan, there is one called 'Victorious,' especially patronized by the damzogs, and that sometimes this instrument resounds when no one is near. This phenomenon announces that some great event is about to happen—some foreign or intestine war."—(pp. 69-73.)

It is much to be desired, now that our modern spirits are beginning to intermeddle a good deal in matters of every-day life, that they would take example from the damzogs, and make themselves generally useful. The physical manifestations of the damzogs, whether musical or general, unlike the incoherent and often inexplicable, if not unmeaning, manifestations of our modern, or as they may be termed from their exhibition through "Mediums," mediumistic spirits, are all devoted to some comprehensible and practical purpose. The mediumistic spirits have not overlooked the importance of giving to their communications with mankind a practical bearing upon the common events of life, but hitherto they have failed most lamentably in their efforts. A continued series of undoubted, immediate perceptible benefits arising from the supposed spiritual aid, would assuredly do more to remove scepticism about the reality of that aid, than the attempted inculcation of contradictory and incongruous systems of morals and philosophy. The examples which are published of assumed spiritual aid in the ordinary business of life are of the vaguest and most unsatisfactory description, insufficient to satisfy a reasonable doubt. The instance already referred to, related by Dr. Hare, as will be seen in the sequel, was the mere creation of his fancy. An instance told by Mr. Home is to the last degree inconclusive. Early in his mediumistic career this gentleman was directed by a spirit to visit the house of a stranger, Mr. B. No reason was given for the direction. After some natural hesitation he obeyed the spirit's behest. On reaching the door of the house to which he was sent, he was seized with a vivid impression that Mr. B.'s mother was seriously ill. The door was opened to him by Mr. B. himself, who was intensely surprised, as might well be imagined, when Mr. Home said to him, "Your mother is ill, and I have been sent to say what will relieve her." Now it appeared that the lady had been taken ill an hour previously, and, so far as can be made out from the story, *after* the time when the spirit had communicated with Mr. Home, who, on entering the house, fell into a trance. While in this state he was conducted to the patient's room. There he made a few passes over her which removed the acute pains from which she was suffering, and he also prescribed certain simple remedies for immediate and others for continued use. "I was then led (to use Mr. Home's own words) by the unseen power into the sitting room, and there returned to my normal state, greatly surprised when these things were related to me. The doctor arrived in about an hour, to find his patient quite out of danger, and, on examining her, he said that from the nature and violence of the attack, it would, in all probability, have been fatal, had steps not been taken at once to alleviate the symptoms. A letter written a few weeks after to a friend, by Mr. B., says that his mother has not had such health for eighteen years past as she now enjoys; she follows implicitly all the instructions given through Daniel (Mr. Home), and the effect is magical!"* It is hard that in so marvellous a case, where every facility existed for obtaining an accurate history, we should be fobbed with so lame a recital; and it is simply inhuman that the world should be deprived, without, apparently, any assignable cause, except one we are loth to mention, of the valuable lesson which might be derived from it. What was the nature, or, at the least, what were the symptoms of the violent and serious malady from which the patient suffered? What were the simple remedies prescribed, which proved so magical in their effects? When points so important, essential to a correct understanding of the story, and embodying the sole aim and end of the spirit's interference, are slurred over in this slip-shod fashion, is it not to be feared that other and equally weighty matters necessary for the right apprehension of the more mysterious parts of the event have been overlooked in the recital? A story that suggests doubts of this nature is manifestly worthless as evidence, such as Mr. Home would have it be. It is a curious fact, also, that at one time Mr. Home proposed to study medicine, although he was then, as he avers, gifted with the power of ascertaining the nature of and healing diseases by supernatural aid. "At Springfield," he writes, "there were many instances of the sick being healed. I was so sensitive to any one who came near me in a diseased state, that I not only myself felt but accurately described their symptoms, and the seats and causes of disease."† Surely the study of medicine under these circumstances would have been supererogatory?—yet Mr. Home went to New York, with the intention of beginning a course of medical studies! He was, however, prevented "by a chain of untoward circumstances from carrying out his intentions," for which, subsequently, he states, "he had occasion to thank God that it was so ordered." It is inexplicable to the uninitiated that a man so marvellously endowed should have thought it requisite at all to study physic. But inconsistencies of this kind are unfortunately the staple of Mr. Home's marvellous experiences. "My health," he says, at another time, "had suffered from the nervous anxiety of my solitary life and studies, and now the medical men whom I consulted pronounced my left lung to be diseased. My spirit friends, said that they were correct in their diagnosis," &c. (p. 49.) Why, of all cases, did the spirits wait for the intervention of the doctors in this particular case, leaving their cherished medium to the doubtful chances and imperfect foresight of human medicine?

---

* *Incidents*, p. 27. † Ib., p. 15.

It cannot well be a matter of surprise that examples of the utility of mediumistic spirits in the ordinary affairs of every-day life, surrounded by so many circumstances engendering doubt, have not carried conviction to the minds of the sceptical. The little facility of mundane interference possessed by the mediumistic spirits, and its inconclusive character, as compared with that of the Darfur and all other Mohammedan genii, are not the least perplexing features of the "spirit-manifestations." It cannot be objected that no just comparison can be made between the two forms of spiritual belief. The Mohammedan belief rests upon a basis as firm as that upon which the mediumistic belief depends. Faith, without logical disruption, cannot be entertained or undermined in the one, without entertaining or seriously damaging the stability of the other. Mr. Howitt manifestly appreciates this position, and admits its reasonableness in his recent work. It is not for us to explain how it happens that so-called spiritual communications take place with more freedom with Mohammedans than with Christians. If, as we are taught, the present influx of mediumistic spirits is an indication of human progression and sublimation, it is obvious that there is some egregious error in the estimate which Western nations are accustomed to form of their advanced civilization as compared with other races. Again, if supposed spiritual communications of the class under consideration are to be regarded as significant of a higher and more spiritualized state of the people subjected to them, Christianity must perforce yield precedence in these respects to Mohammedan and other pagan nations. These difficulties, however, are not insuperable, for we are taught, in communications from the mediumistic spirit-world, that although Western races have made great intellectual advances, they have long been dead morally, and that Mohammed and certain ancient pagans of note occupy higher places in the spheres than Christ himself. Further, from a communication furnished by the spirit of Thomas Paine, in continuation of, and consistent with one from (we hesitate to write the name, but it is necessary for the full elucidation of the subject with which we are engaged) Jesus Christ, on the nature of the Spheres, we learn that "it matters not what may be the name of the God we worship. He may be called Jehovah, Jupiter, or Manito. It is the *way* in which we worship HIM that is of real importance." This removes many embarrassments which interfered with the classification of all the so-called spiritual manifestations of the character under consideration, whether pagan or Christian, in one category. The conception that Christian nations have lagged behind pagan in their spiritual receptivity, will, therefore, excite no surprise. A

difference is even observed between Christian nations in this respect. England, for example, is less susceptible than America. From these considerations it would follow that the facility of spiritual manifestation is in proportion to the receptivity of a people. In England the physical manifestations have been by no means equal to those observed in America. "In the English atmosphere and the English mind," says Mr. Howitt, "the spiritual agency has met with a more resistant principle than in the New World. Probably our denser atmosphere, less electrical and magnetic in its character, and our different telluric conditions, are less favourable to the transmission of spiritual impressions, but more probably the chief obstruction lies in the indurated and materialised tone of the English mind. The Americans are conspicuously a more nervous and excitable people than we are. They have grown up rapidly under new climatic influences, new blending of blood and international idiosyncrasies. They have shown a singular genius for mechanical inventions, and an audacity approaching to rashness in inaugurating new social schemes, new religious organizations, and vast plans of political dominion. Their minds, like their institutions, have shot up with a rapidity of growth resembling that of tropical jungles, and have in consequence greater openness and receptivity."—(v. ii. p. 216.) No doubt the spirit of Joshua, the son of Nun, was somewhat infected with the "audacity" referred to when, in a communication "On the Starry Heavens," made on the 24th of April, 1861, explaining the cause of "this power of the spirit" being *first* manifested among Americans, he said, after schooling them on the riot and licentiousness into which their freedom and independence had degenerated, and the present and prospective disasters of the fratricidal war they were then entering upon:—"But your land, oh Americans! will lead the way in the van of progress. You will be pioneers of this new *régime*. You who have been favoured with the first spiritualistic teachings will be the first to show the world the beauty of the results they bring about in man. You will be the first to show the nations an instance of a country governed without rulers—truthful, loving, and holy, without an established church, and so purified from sin as to need no physicians. This blessed and happy state of your future will be brought about by sad, sad suffering and sorrow; but when disease has entered deeply, the remedies must, of necessity, be severe also."*

These observations, unhappily, throw us back once more into a difficulty which Mr. Howitt's remarks and Mr. Paine's

* *Essays on Various Subjects,* p. 146.

new teachings had very nearly helped us out of. From these teachings it was just to suppose that the less perfect physical developments of the mediumistic spirits arose from the lesser degree of receptivity they encountered among Christian nations —less as compared with the previous condition of those nations in the so-called *dark* ages, as well as with heathen nations at the present time. But the authoritative declaration of Joshua that America is the *first* nation favoured with the spiritualistic teachings derived through the medium of physical manifestations, and that to her all nations will owe their spiritual purification, once more casts a doubt over the question.

But be this as it may, so far as the spirits have succeeded in displaying corporeity they have succeeded most admirably. When, however, spirits become capable of projecting themselves, or any portion of themselves, in so solid a fashion that muscle, bone, and tendons may, as we have already seen, be distinguished, it is certain that we ought to possess some satisfactory test by which we may detect them, or such fragments of them as may be intruded upon us, from ordinary human beings, or from the extremities of such beings. And this, which is true of the physical, is also true of all other manifestations of a *quasi* spiritual origin. Here our ghost-seers would do well to take a practical hint from the experience of their Eastern brethren. The Persian or Anatolian, if by chance he is thrown into contact with an individual whose humanity he questions, possesses a ready method of solving the doubt. He has but to seize a portion of the dubious person's habiliments in such a fashion that the middle finger of the hand is on the one side, and the first and third fingers on the other side of the portion grasped, when, if the mistrusted individual be a spirit, he will at once assume a form more consistent with his supernatural character, or he will incontinently vanish. Doubtless, the greatest of modern magicians, M. Eliphas Lévi, would be able to explain the mystery of this potent grasp. It is not to be inferred, however, that the grasp would prove equally efficacious in eliciting the true character of occidental spirits when they have assumed tangible qualities. The Oriental test refers to a much higher grade of spirit projection than has yet been witnessed here, and for aught that is known to the contrary, may be influential over Mohammedan ghosts alone. All that is sought to be conveyed by the illustration is the need of some test suitable to the present state of manifestations of our Western ghosts, which would enable persons of ordinary mental capacity to be satisfied beyond the reach of a reasonable doubt that these manifestations are such as they are averred to be. In the instances of spirit-projection previously referred to, *invisibility* of the spiritual hands and arms was chiefly relied upon as the criterion of their supra-mundane character. But the whole of the manifestations took place in the *dark*, or in a more deceptive twilight, and it is not clearly explained how visibility was to be looked for under such circumstances. Not that spirits are altogether dependent upon extraneous sources of light for luminousness. The popular domestic ghost—the prescriptive ghost *par excellence*—is principally characterized by its self-luminous qualities. In two or three instances recorded in Mr. Home's autobiography, it is stated that the spectral limbs were luminous, but the accounts are vague, and the phenomenon was by no means certain in its manifestation (pp. 142, 192). In one case where a "black "shrivelled hand arose," on some of the spectators remarking that they could not see it very well, the spirits considerately drew the curtains back from the window, and raised the blind, so as to admit more light from the moon and neighbouring street gas-lights. The spectral hand engaged in picking Mr. Wilkinson's pocket, as already related, was described by that gentleman's daughters as having "phosphoric fingers." The same expression very aptly characterizes certain luminous phenomena which have distinguished some of Mr. Home's later manifestations. For example, in a sitting on the 19th March, 1860, Mr. Home was led, by the spirits be it understood, to the end of the room, "*which was very dark;* he was raised from the ground, a beautiful star was visible, and also one like a small comet. He *said* a star was on his forehead, and one on each hand; we saw the three very bright, and many others glancing about. He was fixed against the wall. The luminous appearance was so distinct as to render the papering on the wall perfectly distinct. . ." Again, of a sitting on the 3rd July, in the same year, we read "He (Mr. Home) then went to the end of the room into the darkness, and we could not see him ascend, but three bright stars were shown which denoted where he was." In another sitting, four days afterwards, Mr. Home "walked about the room, led apparently by a spirit, a very large bright star shone on his forehead, several clustered on his hair, and on the tips of his fingers." It is curious to contrast the certainty with which these luminous phenomena occurred with the doubtful and rare self-luminousness of the spectral limbs. Now it is an important fact, which it is well to be aware of in estimating the value of the foregoing phenomena, that *phosphorus* is a recognised means of acquiring luminous properties among the spirits! Mr. Howitt relates the case of a farmer named Koons, living in an out-of-the-way place among the Ohio Mountains, who was peculiarly favoured with spirit intercourse. He built a wooden hut for the special reception of his *quasi*-immaterial visitors, who were much devoted to daily musical exercitations. Large provision was made for the indulgence of this inclination, and the spirits were accustomed to play so energetically and powerfully in concert upon the instruments set apart for their use, that the performance would prove to be positively alarming to the listeners. A "strange, unearthly voice" at times accompanied the music; and instruments would become endowed with motion. A Professor Mapes, who visited Koons' rooms, and was present at one of the performances of "the strange spirit-bound play," says, "The whole house was on a jar and vibrating in perfect time with the music: and I knew no mortal hands held the instruments." The performance, as was to be anticipated, was carried on with closed shutters, that is to say, in the dark. The rest of the story must be told in Mr. Howitt's words: "Koons handed to the spirits [as the final act of the play, it is to be presumed] some phosphorus in a bit of paper, with which the tambourine-player rubbed his hands, so that they became visible, and could be seen whirling the tambourine over their heads with a rapidity, here and there, like flashes of lightning, or more like the flickering of a light thrown from water on a mirror, than anything else. The shining hand made blows, causing a sensible rap where it touched, and also wrote them a long letter. The visitors all shook hands with the spirits" (p. 192). The use of phosphorus in conjuction with admitted "spirit-manifestations" of high tension is exceedingly instructive.

Roughly formularized, it may be said that the self-luminousness of the mediumistic spirits is in the inverse ratio of their corporeality. At least this is the conclusion forced upon us by Mr. Home's experiences. When the spectral projection became such that it was undistinguishable by the touch from living substance, invisibility was the almost constant concomitant. When the spectral limbs or the spectres themselves became visible, they commonly possessed the characteristics of the ordinary apparition of domestic life. See, for instance, Mr. Wilkinson's account of the "delicately beautiful female hand and part of the forearm, apparently of ghostly tenuity;"* and the relation of the sitting in which Mr. Home was led "to the further end of the room," by a form, "over the head of which was thrown a tinted robe flowing to the ground, marking the head and shoulders." (p. 202.)

A typical example of the common ghost of northern climates —we say common because ghosts can very conveniently be divided into two great categories, namely, the common and the proper; in the former of which may be included the bulk of

---

* *Incidents*, p. 81.

those illusions which foster the more popular forms of ghost-belief: in the latter those graver and more deeply-seated disturbances of the senses, hallucinations, both categories coming under the generic head of prescriptive ghosts—a typical example of the common ghost is afforded by the famous apparition of Marley. The body, it will be remembered, was transparent, so that when Scrooge looked at the phantom of his deceased partner he could see, through the waistcoat, the two buttons on his coat behind. And here may be commended to all ghost-seers the admirable mental disposition in which Mr. Scrooge set himself to contemplate Marley's ghost. The scene in which it is brought to light should always be present to the mind of every one who may be thrown (and who may not?) into contact with ghosts. Our ghost-seers would profit mightily if they would but summon the phantom of Mr. Scrooge to their aid. A few seconds with the spirit of this worthy gentleman would prove more beneficial to themselves and the world generally than hours of pseudo-scientific gossip, and volumes of communications from the spirits of Franklin, Cuvier, Galileo, Galen, and the whole host of physicists from the spheres, judging from the teachings hitherto vouchsafed by these worthies. "'Can you—can you sit down?' asked Scrooge, looking doubtfully at him (Marley). 'I can!' 'Do it, then!' Scrooge asked the question because he didn't know whether a ghost so transparent might find himself in a condition to take a chair; and felt that in the event of its being impossible, it might involve the necessity of an embarrassing explanation. But the ghost sat down on the opposite side of the fireplace, as if he were quite used to it. 'You don't believe in me,' observed the ghost. 'I don't,' said Scrooge. 'What evidence would you have of my reality beyond that of your senses?' 'I don't know,' said Scrooge. 'Why do you doubt your senses?' 'Because,' said Scrooge, 'a little thing affects them. A slight disorder of the stomach makes them cheats. You may be an undigested bit of beef, a blot of mustard, a crumb of cheese, a fragment of underdone potato. There's more of gravy than of grave about you, whatever you are!'"

We would invoke the spirit of Scrooge in reviewing the chief evidences advanced in favour of the recent "spirit-manifestations." The main difficulty experienced in dealing with gregarious ghosts is that of elucidating satisfactorily the sources of wilful or involuntarily deception. There is no variety of the ghost prescriptive—visual, auditory, gustatory, or tactile—which may not be produced by collusion or self-deception. Nay, in the present day, the art of ghost fabrication has attained a pitch of excellence which almost casts the original phantoms into the shade. Witness those admirable stereoscopic photographs, by means of which ghosts of Marley's type have become familiar to most persons, without the need of experiencing the uncomfortable sensations peculiar to a genuine apparition. Singularly enough, our modern spirits, not to be outdone, and as their latest and most novel physical manifestation, have resorted to the photographer—with very indifferent success, it must be confessed. Witness, moreover, and in an especial manner, Mr. Dirck's patent ghost, by means of which, and under the agreeable guidance of Professor Pepper, the public may make acquaintance with the characteristics of the more highly developed class of ghosts which, as in hallucinations of vision, present to the sight the aspect of a real, substantive existence. Mr. Dirck's ghost is paraded daily at the Polytechnic Institution, and it is a triumph of art. The process by which the ghost is produced has not yet been made known, but the secret will be unfolded in due time. The ghost, indeed, is *patented!* Truly a patent ghost is the legitimate complement of ghosts so material as to possess bone and muscle. Until the patent restriction is removed, it would be a waste of words to speculate upon the secrets of the prison-house from which Mr. Dirck's ghost emerges. Let Professor Pepper make known the story when the proper time comes. In the meantime, however, the curious may find many useful hints on the production of ocular spectres in Sir D. Brewster's "Natural Magic." By the patented process a spectral figure of an animate or inanimate object is projected on the stage, the outline being so well defined and the solidity of aspect so real in appearance, that but for its greater illumination it would not be very easily distinguished from actual objects in the vicinity. Offering no greater resistance than the air to the passage of a solid substance, the spectre, nevertheless, shuts out as completely as an opaque body whatever lies behind it. It is an interesting fact, however, that on account of this seeming solidity, the ghost in a great degree fails to impress the spectators with a full sense of the supernatural — inseparable from the popular conception of a spectral appearance as "a thin, sheeted phantom." Gazing upon Mr. Dirck's ghost, the thrill of "grateful terror," which an apt representation of the common domestic apparition never fails to evoke, is altogether missed. For the rest of the prescriptive spectral phenomena let the feats of Houdin, Robin, and their congeners speak. Houdin's Memoirs,* it may be observed, is a highly instructive

* *Memoirs of Robert Houdin, Ambassador, Author, and Conjuror.* Written by Himself. 2 vols. London, 1859.

book for all those who would learn in what manner erroneous judgments are liable to be formed on observed physical phenomena, and the senses prove traitors, and who have not the opportunity of being trained in the severe school of experimental science.

Mr. Howitt has conclusively shown that prescriptive spectral phenomena and the recent "spirit-manifestations" are identical in character, the differences observed chiefly arising from the fact, that whereas previously these manifestations were disconnected and irregular, they are now connected and systematic. It will not excite surprise to learn, then, nay, it would follow as a necessary consequence, that the mediumistic, as the prescriptive phenomena, may be counterfeited. Of this fact not a doubt can be entertained, for it does not rest upon asseverations of the sceptical, but upon the spiritually-inspired statements of Mr. A. J. Davis, the celebrated Poughkeepsie seer, an acknowledged apostle of the mediumistic faith. He was instructed from the spheres that *six* per cent. of the supposed spiritual phenomena observed in America was due to voluntary deception, and *sixty* per cent., including the instances of wilful fraud, to material causes. We shall have occasion again to refer, and in greater detail, to this statement. It is alluded to here in order to show that the question of imposture, or of material agency, must be kept prominently in view in considering the phenomena assigned to spiritual agency. The chief issue arises with regard to the residuum of phenomena, whether mediumistic or prescriptive, which manifestly, or presumably, does not arise from deceit, whether wilful or involuntary, and on the reality of which no doubt can, or, it is averred, ought to be entertained. On the one side it is asserted that these phenomena are of supra-mundane origin, and, if rightly understood, necessary to the perfection of man's moral salvation. On the other side it is maintained that they are of mere mundane origin, and, if rightly understood, their highest utility is restricted to man's physical salvation. "The ghost proper," Professor Pepper teaches, in effect, is most useful as a forewarning of mental or physical disorder, and if justly heeded may save the ghost-seer's bodily or mental health, nay, even his life. It points to the doctor's consulting-room, not to the spheres; to decay, not regeneration; to lunacy, not inspiration; to Hades, not Elysium; to physic, not to nectar." The "spirit-manifestations," Mr. A. J. Davis argues, point to a third dispensation, granted as a coronation of the Christian law. "If the Christian dispensation is an endorsement and fulfilment of the Mosaic law, why (he asks) may not a *third* dispensation come *as a coronation* of the Christian law . . . . ?" The "spirit-manifestations," urges a more orthodox medium, are the forerunners of the true millennium, when Christ himself will appear among us, and angels will walk visibly and talk with men ;"* and when men " —all their impurities having been cast aside—their garments washed—their spirits purified, and their souls raised to an exalted and ennobled condition, shall be enabled to pass from earth to heaven, and scarcely know the change. Earth, so corrupt, so fallen, so degraded by the sins of her people, shall, once more, raise her head in renovated beauty. Her animals, her plants, her flowers, her fruits, shall be spiritualized and purified. Men and maidens shall walk as angels among them, and there shall be no discord, contentions, famines, pestilences, or battles—all shall be peace, harmony, love, joy, and rejoicing. This sphere shall only be the prelude to another and higher, 'and they shall not hurt or destroy in all my holy mountain.' "†

These are the alternatives submitted to us. Either one is weighty, but the supra-mundane is incomparably more so than the mundane. Let us, then, under the guidance of Messrs. Howitt and Home, endeavour to ascertain which is the most probable—if indeed, in pursuing the inquiry, we do not find, so far as the "spirit-manifestations" are concerned, that the very ground of inquiry recedes further and further from us the more we push the investigation.

"Spiritualism" is the term which has been adopted to express the theory which has been based upon the so-called "spirit-manifestations" by those who ("spiritualists," to wit,) believe in their supernatural character. It is a term that had long been in use to designate an ontological hypothesis, the supporters of which were in like manner termed spiritualists. Spiritualism is, indeed, one of the synonyms of immaterialism. The adoption of a well-known and accepted term to express certain novel phenomena, or opinions founded upon the phenomena, having no necessary connexion with the term adopted; to those who are not accustomed to weigh the legitimate signification of words, or the influence they exercise over the reasoning powers, gives a seeming credibility to the asserted phenomena and the opinions sought to be attached to them. To use words having a received and well-understood meaning in another and very different acceptation, necessarily awakens the gravest suspicion either of the mental capacity or the integrity of the user. Whether the adoption of the term "Spiritualism" by the "spirit-rappers" is simply a verbal fraud, or a result of ignorant presumption, must be gathered from the sequel. Hitherto we

---

have avoided using the term in the sense in which it is employed by the mediumistic brethren, but this abstinence is no longer possible when reviewing any of their works, from its frequent recurrence in them.

The new and more general appearance of Spiritualism in the Western hemisphere originated, Mr. Howitt tells us, "in the ordinary visit of what the Germans had denominated a *Poltergeist*, or knocking-ghost; but either the temperament of the North-American public was more favourable to its rapid development, or the time had come, in the general scheme of Providence, for a more full and decided prevalence of spiritual action; for it spread with almost lightning rapidity, assumed new and startling forms, and speedily established itself as a great and significant fact, in the convictions of more than three millions of people of all classes, professions, and persuasions." The origin of the American movement, as is well known, took place in the family of a Mr. John D. Rochester, a farmer, living at Hydesville, County Mayne, State of New York. Mr. Howitt adopts the account of the origin as related by Mr. Robt. Dale Owen,* and he records the most noteworthy part of the history in the following words:—

"The neighbours being called in by the Foxes on this memorable night of the 31st of March, 1848, (when the intelligence of the knocking agent was first discovered), grew to a crowd of seventy or eighty persons. Numbers of questions were put to the spirit, which replied by knocks, that it was that of a travelling tradesman, who had been murdered by the then tenant, John C. Bell, a blacksmith, for his property. That his name was Charles B. Rosmer, [Mr. Owen spells the word Rosma], and that his body had been buried in the cellar by Bell. The servant girl living with the Bells at the time, Lucretia Pulver, gave evidence that she had been suddenly sent away at the time the pedlar was there, and sent for back afterwards; had found the cellar floor had been dug up, and that Bell afterwards repaired the floor in the night-time. The pedlar had never been seen afterwards; and on the floor being dug up to the depth of more than five feet, the remains of a human body were found." (ii., p. 174.)

It will be conceded that the evidence in support of any given facts should be accurate and unassailable in proportion to the importance of the consequences to be derived from them. It would be difficult to overrate the momentous character of the knockings heard at Hydesville, if the estimation in which they are regarded by the "spiritualists" be correct. What then is

---

* *Footfalls on the Boundary of Another World.* With Narrative Illustrations. By Robert Dale Owen, formerly Member of Congress, and American Minister to Naples. Philadelphia, 1860.

the nature of the evidence upon which the supernatural origin of these knockings is chiefly based? It is not necessary to extend the examination beyond the facts quoted from Mr. Howitt. They were of a character which could readily be submitted to independent confirmation, and thus removed from one of the most serious sources of error. Now, the first fact which rivets the attention is this: the name of the murdered pedlar was originally given as "Charles Rayn"* not "Charles J. Rosma." The "remains of a human body" rest upon no higher evidence than this which follows, as stated by Mr. Owen:—In the summer of 1848 (previous researches, it is said, having been foiled by an excess of water beneath the surface, arising from a neighbouring stream in a state of inundation) on digging in the cellar to a depth of five feet, a plank was come to. Digging further, several pieces of crockery and some charcoal and quicklime were found, "indicating that the soil must at some time have been disturbed to a considerable depth;" and finally there were discovered "some human hair and several bones, which on an examination by a medical man skilled in anatomy, proved to be portions of a human skeleton, including two bones of the hand and certain parts of the skull; but no connected skull was found."† Contrast this vague and inconsequent account of the evidence, and the evidence itself, of a fact upon which it is asserted the future destinies of the human race here and hereafter will largely depend, with the evidence sought for and acquired, and the mode of acquisition, in the infinitely less important discovery of an ancient jawbone at Abbeville! Of the depositions in the case Mr. Owen frankly tells us that "Nothing positive can be gathered from them."‡ Moreover, he, an admiring believer in the manifestations, writes:—

"It is proper also to state in this connexion, that a few months afterward—to wit, in July or August, 1848—a circumstance occurred at Rochester, New York, somewhat analogous in character, and indicating the danger of indulging, without corroborating evidence, in suspicions aroused by alleged spiritual information. A young pedlar, with a wagon and two horses, and known to be possessed of several hundred dollars, having put up at a tavern in that city, suddenly disappeared. Public opinion settled down in the belief that he was murdered. An enthusiastic Spiritualist had the surmise confirmed by the raps. Through the same medium the credulous inquirer was informed that the body lay in the canal; several spots being succes-

---

* *Sights and Sounds, the Mystery of the Day: comprising an entire History of the American Spirit Manifestations.* By Henry Spicer, A.M. London, 1853. p. 59.

† *Footfalls*, p. 293. ‡ Op. cit., p. 29.

sively indicated where it could be found. These were anxiously dragged, but to no purpose. Finally, the dupe's wife was required to go into the canal at a designated point, where she would certainly discover the corpse; in obeying which injunction she nearly lost her life. Some months afterwards, the alleged victim reappeared: he had departed recently for Canada, to avoid the importunities of his creditors.* In the Hydesville case, too, there was some rebutting evidence. The raps had alleged that, though the pedlar's wife was dead, his five children lived in Orange County, New York; but all efforts to discover them there were fruitless. Nor does it appear that any man named Rosma was ascertained to have resided there. It remains to be added that no legal proceedings were ever instituted, either against Mr. Bell, in virtue of the suspicions aroused, or by him against those who expressed such suspicions. He finally left the country. *It is evident that no sufficient case is made out against him. The statements of the earthly witnesses amount to circumstantial evidence only; and upon unsupported ultra-mundane testimony no dependence can be placed.* It may supply hints; it may suggest inquiries: but assurance it cannot give."

Mr. Owen adds:—

"The Hydesville narrative, however, as one of unexplained disturbances, like those at Cideville, at Ahrensburg, at Slawensik, at Epworth, and at Tedworth, rests for verification on the reality of the phenomena themselves, not on the accuracy of extrinsic information alleged to be thereby supplied."†

Such are Mr. Owen's statements and conclusions upon a series of facts which Mr. Howitt relates as if they were beyond the reach of cavil. Setting aside the awkward fact of a presumed denizen of the other world being doubtful of its own identity, we are required to believe of the Hydesville events, that the statements of a spirit as to its own spirituality may be received without question, although that spirit is a convicted liar and slanderer of the very worst grade; and that evidence which it is acknowledged is not worthy of the slightest credence in mundane affairs, may be regarded as more than sufficient in supra-mundane. Whether there is not a more reasonable and natural assumption within reach, which would meet the necessity of the case, is a question which the facts may be left to determine for themselves. Mr. Spicer remarks that we cannot "justly blame the man who admits indifferent premises, for the

* "If we concede the reality of the spirit-rap, and if we assume to judge of ultra-mundane intentions, we may imagine that the purpose was, by so early and so marked a lesson, to warn men, even from the commencement, against putting implicit faith in spiritual communications. It is worthy of remark, however, that there is this great difference in these two cases, that the Hydesville communications came by spontaneous agency, uncalled for, unlooked for, while those obtained at Rochester were evoked and expected."

† Op. cit., p. 297.

sake of securing the widest possible scope to the argument he mistrusts."* Doubtless it is upon some such principle of reasoning as that in regard to which Mr. Spicer offers the foregoing apologetic observation that "Spiritualism" refers its modern renovation to the Hydesville events. But positive and hard lying is not a peculiarity of the Hydesville spirit. Untruthfulness is one of the most marked characteristics of the spirits who have the greatest facility of intercourse with man, those of the "lower spheres." "They give," said the spirit of Thomas Paine, in the communication already referred to,† "what the persons who show them, or the *medium they serve*, may require; and *they often glory in misleading or perverting the truth.*"

England, Mr. Howitt points out, narrowly escaped the honour of initiating the great "spiritual" reform. "The gentleman at Tedworth, who put a direct question regarding the drummer," he says, speaking of the celebrated disturbances at Mr. Mompesson's house, "and was directly answered by affirmative knocks, was on the very threshold of discovery, had he pursued his inquiries. But the following out was left to American acuteness." No doubt; and if it be assumed (the fiction of the murdered pedlar being admitted) that the spirit of Samuel Slick, Esq., of Slickville, Conn., was the active agent in the events at Hydesville, all the difficulties which beset the early origin and subsequent progress of the "spirit-manifestations" in America and elsewhere vanish. The thorough knowledge of "*human natur*," in the sense in which Mr. Slick used that term, exhibited by the spirits from the commencement of the manifestations is inexplicable, except on the supposition that the immortal Clockmaker inspired the movement. But, most singularly, Mr. Howitt does not refer to the Cock-lane ghost. This was a *rapping* ghost, with which an intelligent correspondence was established, precisely after the fashion of the earlier communications with the Hydesville ghost. The Cock-lane ghost, as the Hydesville, professed to be the spirit of a murdered person, and fixed a charge of murder on a previous inhabitant of the haunted house. The Cock-lane ghost did not lack, any more than the Hydesville, its supporters in the shape of "most respectable," "highly intelligent," and "responsible" men. As in the Hydesville ghost, so in the Cock-lane, the statements of the presumed spirit were proved to be a malicious and impudent slander. If the Hydesville ghost had been subjected to tests similar to those to which the Cock-lane ghost was subjected, there can be no

* *Sights and Sounds*, p. 21.

† *Essays*, p. 100.

doubt that the former, as the latter, would have been ascertained to have been an audacious imposture. Finally, if Mr. Bell, the asserted murderer in the Hydesville case, had acted in the same way as Mr. Kempe, the asserted murderer in the Cock-lane case, we should doubtless have heard little more of the American ghost. Mr. Kempe indicted Parsons, the occupier of the haunted house in Cock-lane, his wife, a girl, the curate of St. Sepulchre's, and a tradesman, for conspiring to injure his character. All were found guilty. The clergyman and tradesman were mulcted in the sum of 300*l.*; Parsons set in the stocks, and subsequently imprisoned with his wife and the girl. These proceedings effectually "laid" the ghost. Probably Mr. Bell was influenced in the course he took by the fact that his fellow-citizens, as Captain Kedgwick kindly pointed out to Messrs. Chuzzlewit and Co., liked excitement, and were not long in "riling up."

The parallelism between the Cock-lane ghost and the Hydesville spirit, together with the events which occurred at Rochester, N. Y., as related by Mr. Owen, are facts which require to be kept prominently in mind in estimating the true character of the Hydesville manifestations. If the Cock-lane ghost was an imposture, it is for the historians of the "spirit-movement" to show that phenomena in every respect analogous, as in the Hydesville manifestations, were not produced by imposture. If the Cock-lane ghost was a genuine "spirit-manifestation," as the writer of the preface to Mr. Homes's autobiography would appear to imply (as we shall see presently), it is inexplicable that Mr. Howitt should have omitted any allusion to this remarkable forerunner of the Hydesville events. If it be said that the subsequent spread and wide extension of the so-called "spirit-manifestations" in America alone suffice to give credibility to the exceptional character of the Hydesville events, it is simply necessary to remark, that such a development is by no means beyond the reach of, or inconsistent with, that American acuteness to which Mr. Howitt refers. Forged ghosts would be as apt to tickle the Transatlantic appetite for excitement as aught else that was peculiarly novel and audacious. "We are a smart people here," remarked the editor of the *Rowdy Journal*, "and can appreciate smartness."

"The spirits having found a mode of making themselves heard and understood, seemed determined to be heard to some purpose," writes Mr. Howitt. Knockings of a similar character to those heard at Hydesville quickly cropped out in several localities; and in the house of a Dr. Phelps, of Stratford, Connecticut (Mr. Slick's State, by the way), the mysterious manifestations assumed a more startling character. The clothes of one of the children, a boy, were cut to pieces, fragile articles, such as glasses and crockery, were dashed to the ground, window-panes broken, and other mischievous freaks enacted. Mr. Howitt describes Dr. Phelps as "a man of the highest intelligence and truth," and states that notwithstanding every facility was offered for inquiry, no one could account for the disturbances on natural grounds. It so happens, however, that no less a personage than the Poughkeepsie seer visited the scene, and he traced the whole of the manifestations to the active agency of Dr. Phelps's eldest son and daughter, and others. But having ascertained this fact, he further "discovered when reviewing the circumstances from his superior condition," that the children were impelled to play the pranks they were guilty of by spirits! And, further, that they were so greatly "surcharged with vital magnetism and vital electricity," that when Mr. Davis heard a quick loud sound under the boy's left foot, "he instantly perceived that his system, like the torpedo eel, had discharged a small volume or current of vital electricity from the sole of the foot!"

There is good ground for believing that the mother, if not the father, was a party to the collusion in this case. So rank an instance of imposture, occurring almost immediately after the Hydesville events, and still passing current with "spiritualists," is highly instructive.

The manifestations were not long confined to one or two localities. They rapidly spread throughout the United States. Fortunately, in their subsequent development they were not so mischievous, morally or physically, as at Hydesville or at Stratford. Presently it was discovered that the manifestations were not shown indiscriminately, but only in connexion with certain persons, who were privileged as the medium through which the peculiar phenomena were exhibited, and who were thenceforth termed *mediums.* Soon other and more marvellous exhibitions took place when these mediums were present. Tables became possessed and bounded like kids; handbells flitted about like sparrows, ringing in their joyful flight; pianos discoursed sweet music without human hands; the sick were healed and the stupid inspired; and, finally, spirits, as the veracious history goes, entered into familiar intercourse with man, circles being everywhere formed to receive their communications. So early as 1852, Philadelphia, Mr. Howitt states, reckoned 300 circles, and in 1853 there were 30,000 mediums in the United States. Writing in 1855, the Rev. W. R. Hayden said, "Eight short years ago, not a single individual in the United States was known as a spiritualist; at that date 2,500,000, at a moderate estimate, profess to have arrived at their convictions of spiritual communications from personal experience. The average rate of increase has been 300,000 per annum."

The converts to the new manifestations included, "statesmen, members of congress, foreign ambassadors, judges of the higher courts, clergymen in great numbers, lawyers, doctors, and professors." Chief among these worthies was Dr. Hare, "the most famous practical chemist and electrician of the United States." At first, as other scientific men, he regarded the manifestations as a mere delusion of the senses. He read Faraday's explanation of table-moving, and thought it convincing. But he was induced to submit the phenomena to experimental observation. "He continued the experiments for two years," says Mr. Howitt, "with indefatigable industry, ingenuity, and care. The details of them may be seen in his work on Spiritualism, *Experimental Investigation of Spiritual Manifestations.* The result was an overwhelming mass of facts, utterly demolishing the Faraday theory. The demonstrations were mathematically correct and precise; first, of a power beyond that of human, or of any known mundane agency; second, of intelligence not derived from minds in the body." (ii. p. 181).

But this was one step only, an important one truly, to the difficulty to be solved. Did this intelligence proceed from disembodied spirits? While pursuing his investigations, Dr. Hare had himself become a medium, and frequently received communications professedly from his father and a deceased sister. Now, as Mr. Howitt tells us:—

"One day, on the spirit calling herself his sister presenting herself at his table, as manifested through raps, he told her he wished her to do him a little service. She replied that she would if it were in her power. He was then on a visit to Cape May, about a hundred miles from Philadelphia, and he requested her to go to Philadelphia and desire Mrs. Gourlay, the medium, to get Dr. Gourlay, her husband, to call at a certain bank and ask the note-clerk a question as to the passing through of a bill, and bring him the answer by half-past three. The spirit promised and was absent for half-an-hour, but had then returned with the answer. Dr. Hare made no other communication to Mrs. Gourlay on the subject; but on his return to Philadelphia, in about a fortnight, he inquired of Mrs. Gourlay if she had received any message from him during his absence. She said yes, and under very extraordinary circumstances. She was receiving a communication from her spirit-mother, when the communication suddenly stopped, and his spirit messenger gave her commission. It was attended to by Dr. Gourlay, and the answer returned to him by the spirit. Dr. Hare then went to the bank, and ascertained from the note-clerk that Dr. Gourlay called on the day named, asked a question and received the answer which had been returned to Dr. Hare by the spirit-messenger. Dr. Hare was thus assured that he had an actual spirit-messenger, and was perfectly satisfied. But other doubts had to be destroyed in him by spiritualism. He had all his life been a determined infidel, disbelieving in God, the immortality of the soul, and in revelation. . . . Having convinced himself, however, of his first error as to spirit, his further inquiries convinced him of the truth of the Christian revelation, and a little time before his death he called on the Judge (Edmonds) and said his sister, who had been dead many years, had come to him, and so thoroughly identified herself to him, as to convince him it was herself and that she still lived. He had reasoned thus—'If she lives, I shall live also, and there is an immortality; if an immortality, there must be—there is a God. But,' said he, 'Judge, I do not stop there, I believe in revelation, and in a revelation through Jesus of Nazareth. I am a Christian.' A grand answer to the *cui bono.*" (ii. p. 182.)

Let Dr. Hare now speak for himself. The work referred to by Mr. Howitt* is introduced to the reader by a Preface which commences in the following manner:—

"*Dr. Hare a true Christian though repudiating for the most part the Canonical Gospel.*—In consequence of communications from the spirit spheres, the author has, since the publication of this work, become reconciled and devoted to Christ, as a great and good reformer, who, through a mediumship as preternatural as the powers of computation displayed by Zera Colburn, acquired that knowledge of the spirit-world which has of late been imparted to Spiritualists and been communicated to the author by his spirit-father, sanctioned by a convocation of spirits.† Consistently, advertisements have been made with Dr. Hare's sanction purporting that he was to prove in his lectures that true Spiritualism and true Christianity were identical.

"This caused an inquiry to be made of him, whether he had found himself wrong in the strictures made in this work upon the teachings imputed to Jesus in the Canonical Gospels. Agreeably to the communications which he has had, these strictures were sanctioned by the Nazarene himself, who has alleged that the doctrines and information respecting the spirit-world,

---

* *Experimental Investigations of the Spirit Manifestations, Demonstrating the Existence of Spirits, and their Communion with Mortals. Doctrine of the Spirit-World respecting Heaven, Hell, Morality, God. Also, the Influence of Scripture on the Morals of Christians.* By Robert Hare, M.D., Emeritus Professor of Chemistry in the University of Pennsylvania, &c. We quote from the fifth edition, 8vo, pp. 462. New York, 1858.

† The spirits of Washington, J. Q. Adams, H. Clay, B. Franklin. W. K. White, W. C. Channing, and others attended this Convocation. Several of these spirits had attended one of Dr. Hare's lectures at Boston, and, therefore, were able to testify from personal experience that his knowledge of the spirit-world was genuine!

furnished through the author's humble instrumentality, are the nearest to those which he aimed to impart, of any that have ever been recorded. In order to avoid collision with the bigotry of the Jews, and the prejudices of the Gentiles, his disciples modified, supplanted, and adulterated his teachings. Never, certainly, were any two doctrines more irreconcileable than those of Moses and Christ," &c.

So much for Dr. Hare's Christianity. Let us now see how the story of the bank transaction fares at his hands. In a supplemental preface to his work, Dr. Hare gives the preliminary details, much in the manner stated by Mr. Howitt, but the conclusion of the story is very different from the original version. He writes :—

"The note-clerk recollected the application to him, but appeared to have considered it as too irregular to merit much attention. Hence the impression *received* by the applicants, and communicated to me, was not correct. But as it did not accord with that existing in my memory, it could not have been learned from MY mind."*

In fact Mrs. Gourlay's impression of the events differed from those of Dr. Hare; the bank-clerk's impressions did not agree with those of either Dr. Hare or Mrs. Gourlay; and the actual events differed widely from the facts as originally related, and as recorded by Mr. Howitt. We are then required to believe the fact of spiritual communication, although all the facts capable of confirmation were unconfirmed, or rather proved to be entirely erroneous. The true explanation is, undoubtedly, that a fantastic imagination of Dr. Hare was pandered to by Mrs. Gourlay after the manner of her tribe.

We have now to estimate the value of those demonstrations which Mr. Howitt designates as "mathematically correct and precise." The volume in which Dr. Hare records his investigation contains no less than 462 pages; but of these the experimental investigation itself occupies but 54! the rest being chiefly devoted to Dr. Hare's speculations on the spirit world, heaven, hell, &c. The scientific value of Dr. Hare's investigation may be gathered from what follows :—His chief experiments were conducted by means of an ingeniously arranged instrument through which a medium could act upon the index of an alphabetic dial, so placed that the face was not visible to the medium. The spirit of an uncle sought to spell its name in full upon the apparatus. "Although the requisite letters (writes Dr. Hare,) were ultimately found, there was evidently some difficulty, as if there was some groping for them with an imperfect light. This

* Op. Cit., p. 16.

has since been explained by my father's spirit. *He alleges that preferably the eyes of the medium would be employed, but, although with difficulty, he used mine instead.*" (§ 164.) Just so: the possibility of self-deception never appears to have entered into Dr. Hare's mind. In the ordinary method of rapping out messages by means of the alphabet, the letters are visible to the medium as well as the inquirer; and the success of the medium depends solely upon the indications afforded by the countenance, or the pencil passed over the letters, or both. A passive countenance and an uniformly moved pencil, renders of no avail the highest acuteness and intelligence either of spirit or medium. When the alphabet is hidden from the medium, as when a dial is used such as Dr. Hare constructed, the medium is left entirely dependent upon the hints received from the sitter's countenance. That Dr. Hare's countenance supplied the needful hints (even if more active aid were not unwittingly to himself obtained from him) in the inquiries he conducted, is demonstrated by his own relation of the experiments. How greatly the difficulties of maintaining a spiritual conversation physically are augmented by the adoption of Dr. Hare's apparatus is obvious. It is not, therefore, surprising to find the spirits (or mediums, as you please, reader) objecting very strongly against the use of such apparatus. "Although (writes Dr. Hare) with a view to convince the sceptical, spirits will occasionally give manifestations when the vision or muscular control of the medium is nullified, it is more difficult for them to operate in this way; however, it is more difficult for some spirits than for others. Those spirits by whom I obtained my best manifestations were interested in my success. Others have refused to aid me in like manner. One, who has assisted me with much zeal, has communicated that he would work my apparatus when arranged for a test; but that, as it caused much more exertion, and, of course, retardation, he advised that the test arrangement should not be interposed when it could be avoided." (§ 165–6). In the end, losing patience, the spirits peremptorily rapped out, "*Let the medium see the letters!*" (§ 169.) A sprightly spirit, named Maria, refused to communicate with Dr. Hare through the apparatus. "*It is impossible,*" she said, "*for a spirit to work your apparatus: I am very sorry.*"

Equally obnoxious to the spirits is the adoption of any mechanism to neutralize the physical action of the medium in moving tables. "The interposition of the plate and balls (a metallic plate resting upon two smooth brass-balls) makes it," says Dr. Hare, "more difficult for spirits to move a table than when the hands are directly applied [Dr. Hare appears to have very imperfectly comprehended Faraday's experiments].

In the latter case, the spirits actuate the hands primarily, and the table or apparatus secondarily; but when the hands are incapacitated from influencing the motion, the spirit has to assail the inanimate matter directly, *assisted only by an emanation* [indicated through the muscles doubtlessly] *from the medium* (!). This we read in § 173; yet, elsewhere, we learn from Dr. Hare that spirits can move tables readily, and weighty ones withal, without the medium touching, or, in fact, approaching them. How, then, does it come to pass that the apparatus should prove so huge an impediment to their physical efforts? It is curious that the impatience of tests by the spirits in Dr. Hare's investigations should be so closely paralleled by the impatience of the mediums. Very early in Dr. Hare's investigation, the spirit of his father "tapped," "Oh, my son, listen to reason!" (The medium manifestly had an apt idea of human nature.) Dr. Hare very properly urged that the experiment was of immense importance, if considered as proving that a spirit was present, and had actuated the apparatus, "affording thus precise experimental proof of the immortality of the soul; that a matter of such moment should not be considered as conclusively decided until every possible additional means of verification should be employed. This led my companions to accuse me of extreme incredulity. The medium, said she, 'should not deem it worth while to sit for me again;' and one of the gentlemen sat himself down by the fireside, declaring me 'to be insusceptible of conviction, and that he would give me up.'" (§ 160.)

Finally, it may be mentioned, that Dr. Hare points out that it was quite consistent for a spirit who had no such powerful motive to communicate with him as his spirit-father and a spirit-friend, to prefer "to find an apology for not actuating his apparatus, rather than to have studied, or sought for the means of surmounting the obstacles." (§ 196.) Alas, for Mr. Howitt's mathematically correct and precise demonstrations!

The further history of the spirit manifestations can fortunately be summed up in the histories of three typal men. According to Mr. Howitt, spiritualism has exhibited three stages; the patriarchal or preparatory, the pagan, and the Christian. These stages have been represented respectively by three typal mediums, Messrs. Daniel Dunglas Home, Andrew Jackson Davis, and Thomas L. Harris. The readers of this Journal are already sufficiently acquainted with Mr. Harris.* Mr. Home is the most familiarly known of the typal mediums in this country, and a new interest has been awakened in his career by the recent publication of his autobiography. This work contains

* See vol. i. p. 1.

little novel, except the facts detailed of Mr. Home's early life. The bulk of the book is made up by the promiscuous republication, without modification, of the accounts of Mr. Home's marvellous powers which have appeared in various journals in England and America. Mr. Howitt describes Mr. Home as—

> "An exhibitor of what are called physical phenomena, but which are spiritual agencies acting on matter. . . . . Mr. Home's mission seems to have been to go forth and do the preliminary work of restoring faith by the performance of these outward marvels. Till that foundation was laid there could be no faith in higher and more psychical efforts. He was the herald of more interior truths. By a remarkable dispensation, like the prophets of old, he was taken from the class which had no power in itself, that all the power might be seen to come from on high." (pp. 201, 2.)

Mr. Home was born a ghost-seer. His mother, a Scotchwoman, was a ghost-seer throughout life, possessing the faculty of second sight. Second sight, it is instructive to note, was also hereditary in the Fox family, in whose house the Hydesville manifestations took place. When Mr. Home was a baby, his cradle was frequently rocked by invisible agency; he suggests probably by "some guardian spirit." At four years of age he had a prophetic vision. This fact is stated on the authority of an aunt. When thirteen he was favoured with an apparition; and at seventeen he became subject to spiritual impressions. About this period of his life a spirit communicated with him orally, and soon after he was visited by "knockings," and furniture began to be moved in his presence without visible cause. He was accused of having brought the devil into the house where he resided, and an aunt seeking to overcome a lively table with the Bible, was lifted up bodily with the table. There is a suspicious resemblance between the phenomena Mr. Home manifested at this period of his life and some of those exhibited by Dr. Phelps's son. Subjected to the accusation of being possessed with a devil, he was consoled by a visit from the spirit of his mother, who addressing him said: "Daniel, fear not, my child; God is with you. Who shall be against you? Seek to do good: be truthful and truth-loving, and you will prosper, my child. Yours is a glorious mission—you will convince the infidel, cure the sick, and console the weeping." From the time of this vision date the manifestations of mediumship through which Mr. Home has become publicly known. He was then in America, where, indeed, he had lived since he was nine years old, and the discovery of the means of alphabetic communication with spirits at Hydesville enabled him at once to display his peculiar powers to greater perfection; but he makes no reference in his Life to the original discoverers of this method of spiritual converse.

Mr. Home would date his first appearance in public life from a newspaper account of a sitting published in March, 1851. In this account a Mr. Hayden's name plays a great part as an investigator of the marvellous phenomena. Was this Mr. Hayden the husband of that celebrated Mrs. Hayden, who was the first medium who visited this country, and whose impudent fraud upon the English public will not readily be forgotten?

In Mr. Home's marvellous powers, then, we behold an epitome of the whole of the phenomena peculiar to ghosts prescriptive. In accepting him we are required to accept also very nearly the entire range of existing popular superstitious beliefs in this country. The grounds of belief in the physical phenomena of the so-called "spirit-manifestations" necessitate, as has already been pointed out, the reception of all cognate beliefs. The "Spiritualists" frankly admit this consequence, but even the field thus opened out for the exercise of their faith is not sufficient to satisfy the voracious appetite they display for the marvellous. The writer of the introductory remarks to Mr. Home's autobiography comes forward as a champion of detected imposture, and boldly takes up the cause of the Cock-lane ghost. He admits that when steps were taken to prevent imposition, the ghost vanished, but he ingeniously suggests that the result "might be owing merely to deranged conditions," which was "held by the sapient committee of investigation as clear proof of imposture." When an argument of this character is advanced in the preface to a book containing incidents such as those which have marked Mr. Home's career, it leads to an ugly suspicion that the writer, having an uneasy consciousness that there was nothing in them inconsistent with human agency, aided by a fertile imagination, is anxious to anticipate the objection that might thence arise by hinting that it would not therefore follow that the incidents were not supernatural. Mr. A. J. Davis, with greater frankness, maintained the supernatural character of the pranks naturally played by Dr. Phelps's children. The writer of Mr. Home's preface is conscious of another weak point in that gentleman's recital. Regard Mr. Home's case, he says, "simply as a curious case in pathology—for himself to describe it in detail may be considered as a useful service to mankind, just as it would be considered for us to obtain the self-portraiture of any other peculiar case of nervous derangement." Putting collusion aside, the question whether Mr. Home does not suffer from some form of nervous derangement, which might well account for the supposed phenomena, has to be set at rest before the question of supernatural agency is discussed. Witch-flights and the aërial levitations of ecstatics are more amply confirmed than Mr. Home's elevations, all of which, with two exceptions, not described in detail, took place in the *dark.** When we witness the exaggeration into which Mr. Howitt's imagination has led him, even with the facts of the "spirit-movement," recorded in clear type from good founts, we are thankful that the Spiritualists have not endowed Mr. Home, and the furniture influenced by him, with powers of flight at least equal to those possessed by the witches of old and their broomsticks. The great high-priest of modern magic, Eliphas Lévi, avers that Mr. Home is a victim of contagious somnambulism,† and he designates all genuine mediums as *medio-maniacs.*‡ Mr. Home describes several facts connected with his "highly nervous organization" and delicate health, which would tend to confirm M. Lévi's statement. We do not purpose, however, to discuss these facts here. Following the course we have adopted throughout this article, we shall seek light upon Mr. Home's asserted powers, as well as upon the general phenomena of mediumship, from a source to which even "Spiritualists" must bow.

Andrew Jackson Davis, the third of Mr. Howitt's typal mediums and the representative of the pagan phase of "Spiritualism," was born of poor, illiterate parents, and was at first illiterate himself, but he inherited from his mother the clairvoyant faculty. As a child he heard voices and saw visions, and his clairvoyant faculty had reached a high state of development five years before the Hydesville knockings occurred. He is the great apostle of the *Progressionist* or Pagan (as Mr. Howitt phrases it) party in the spiritualistic movement, a party which, in his own words, "will acknowledge no central mind, no 'control' external to spirit, no outward authority, no allegiances to religious observances, no reverence for past religions, no vicarious atonement for personal sins, no fear of existing statutes, no sympathy with institutions that claim to be superior to the immortal mind of man."§ Of this medium, Mr. Howitt says:—

"His clairvoyance was advanced into clairscience. He beheld all the essential natures of things; saw the interior of man and animals as perfectly as their exterior; and described them in language so correct that the most able technologists could not surpass him. He pointed out the proper remedies for all the complaints, and the shops [? 'we are a smart people here'] where they were to be obtained. The light of all nature appeared laid before him, and he saw the metals in the earth like living flames, and lights and flames emanating from every portion of the living structure of men and animals. The most

---

* *Incidents*, p. 39. † *La Clef des Grands Mystères*, p. 193.
‡ *Histoire de la Magie*, pp. 446, 491, &c.
§ *Progressive Annual for* 1863. New York: A. J. Davis and Co. p. 7.

distant regions and their various productions were present before him." (ii. p. 205.)

Here, then, we obtain a guide to the mysteries of mediumship whose authority cannot be questioned. Mr. Davis describes twenty-four varieties of mediums.* Some of these only need occupy our attention. And first of the *motive* medium, of which variety Mr. Home is indicated as a conspicuous example. "There is none more pregnant," says Mr. Davis, "than the simple motive medium with *puerilities and incongruities,*" but he adds, "and with startling phenomena also." But when, as in the case of Mr. Home, "the impersonating, sympathetic, the clairvoyant, and the impressional attributes of mediumship are added, as they are under the most favourable circumstances, the facts being well authenticated, such a person may be called a *compound* medium. And yet, with this combination, unless the strictest care be preserved during the sessions, the medium will be confused with *wrong* sounds, visions, and communications, which, because the medium is not in the Superior Condition (which brings wisdom to the mind), he may charge in full confidence to extraneous causes—usually, to the undeveloped evil or inconsiderate condition of the spirits."—(Op. cit., p. 133.) Of the *impersonating* medium we are told that "there is a strong probability always that the representation may be shaded and biassed by the mind of the medium, wherefore it is seldom reliable."—(p. 137.) The *sympathetic medium,* we learn, is apt to fashion his communications by his own peculiar leanings and prejudices; and is "open to every modification of contradiction."—(p. 151.) *Impressional* media are the source from which is now flowing into the world "a mass of literature—a strange combination of prose, and so-called poetic verbiage—which it seems to me the world might easily progress without." —(p. 196.) Finally, we are given to understand that the *clairvoyant* medium is not altogether dependent upon the spirit-land for revelations of truths or perceptions of great thoughts, and "may be hallucinated and completely deceived by the insinuating presence of some psychological influence."—(p. 192.)

Mr. Davis further instructs us that of the phenomena of mediumship six per cent. are due to voluntary deception, fifty-four per cent. to certain material influences which he sums up under the different heads, "neurological," "vital electricity," "nervo-psychology," "cerebro-sympathy," and "clairvoyance;" and but forty per cent. to "departed spirits." Moreover, in a disquisition on the causes of error in the manifestations we are

---

* *The Present Age and Inner Life; a Sequel to Spiritual Intercourse. Modern Mysteries Classified and Explained.* p. 130.

taught that, "It requires on the part of the medium, the seer, the prophet, the mediator, &c., a liberal amount of psychological education and experience, in order to be able, with any degree of truthful discrimination, to detect the difference between impressions received from minds in this world and those which emanate from the higher sphere."—(p. 202.) Again, it is said: "The spiritual cannot, as a general principle, enter into human or terrestrial media or receptacles, without partaking, like water, of the shape of the vessel into which it is poured."—(p. 203.) Hence we are not surprised to learn that, "*it is not safe*," for mediums to rely upon supposed or possible spiritual impressions, "*without the entire approbation of their own judgments and powers of understanding upon what may be thus communicated,*" (p. 204) :—a caution much needed by spiritual writing media, who, "*while absorbed in the act of writing*, may not be conscious of lending any involuntary assistance to the process, nevertheless there stands, inflexibly, the everlasting possibility of a fusion or confusion, or admixture of the thoughts of the writer with the spiritual dictations."—(p. 204.) We are prepared then to read, as the sum of the whole matter, that—"IT IS AN UNWARRANTABLE THING TO LOOK FOR PERFECT WISDOM, OR FOR INSTRUCTION MUCH SUPERIOR TO THE MENTAL DEVELOPMENT OF THE MEDIUM."—(p. 72.)

Here, then, we have it answered in effect by the greatest of modern seers, a seer who is stamped as genuine by Mr. Howitt himself, that the knowledge of the medium is the limit of the supposed spiritual knowledge vouchsafed through him. This removes all difficulties out of the way of a right comprehension of spiritualism and spiritualists. We now learn how it is that whatever has outraged common sense on earth, whether in religion, or science, or morals, receives a species of apotheosis at the hands of the spiritualists. Mr. Howitt discovers much of genuine gospel in Mormonism. Homœopathy, mesmerism, odylism, and the whole range of pseudo-sciences, have received a celestial endorsement. We are not at a loss now, when Mr. Davis describes and depicts from visual observation, the phenomena of table-moving as being effected by a *lever* made from a "fine thread of magnetism," worked by spirits; or when this gentleman accounts for a little expectorated blood, by stating that it was emitted by the capillary vessels of the brain! We also can now understand how it is that the spirits are liars, illiterate, contradictory, oft debased; how it comes to pass that heaven is an imperfect copy of earth; that there, as here, are required schools, places of religious instruction, missionaries, mechanics' institutes, and societies for mutual improvement; how the highest spirits are addicted to cardplaying, and indulge in bull-fights* and field sports ;† how even a currency, and *quasi*-mundane clothing, even to gloves,‡ are required in the spheres; how departed spirits indulge in the same partialities above as they gave way to below (Franklin, for example, is at work perfecting some electrical apparatus, and not long ago he appeared to a medium with an electrical machine under his arm, to aid in communicating with his earthly friend). But why multiply the record of these absurdities. Let it suffice that Christ himself is represented as saying: "Man lives in the spheres with all his tastes and fancies. If he could not gratify them it would be no happiness to him to be there!"§

But the fact of spiritual intercourse by means of the so-called "spirit manifestations," it may be said, remains. Let Mr. Davis resolve all doubt on this subject, and on such "startling phenomena" as he may have referred to when writing of motive mediums. An instance from his account of the disturbances in Dr. Phelps's house will prove sufficient for our purpose—disturbances which rank among the gospel events of "spiritualists."

"The parents of Henry," he writes, alluding to Dr. Phelps's son, "I believe have received his testimony as being literally true, but I have discovered that he frequently failed to discriminate; on one occasion he was found with a rope passed under his arms, and suspended to the limb of a tree. When removed from that position he related that he 'screamed at the top of his voice,' but it was ascertained that had he in reality done so, the domestics who were near the spot must have heard him. Now it was not with the intention to deceive that he made this declaration; he really supposed that he had called aloud, as I discovered when reviewing the circumstances from my superior condition, at which time I also learned that to control the boy from effecting a premeditated imprudence, a spirit near him, taking advantage of the electrical state of his system, actually made him unconsciously instrumental in *tying himself* to the tree, and in order that he might not escape and accomplish his previously conceived design, the guardian spirit impressed him to feel fright, and to *think* that he called for help till such time as was deemed prudent to release him."||

This needs no comment. The question we proposed to solve has not as yet, in respect to the "spirit manifestations," any place. It may be that somewhere, buried deep beneath the farrago of folly and blasphemy we have detailed, there is a small nucleus of honest,

---

* Cahagnet, *Revelations d'Outre-tombe*, p. 259.

† Cahagnet, op. cit., p. 302. ‡ *Incidents*, p. 81.

§ *Essays*, p. 88.

|| It is to be feared that Mr. Davis has a personal interest in this style of reasoning. He appears to have been partial to plagiarism, the matters plagiarized being given by him as celestial communications. Doubtless he would say that he was "impressed" by spirits to this literary pilfering. See Cahagnet, *Revelations d'Outre-tombe*, p. 9. *Spirit-rapping*. Vizetelly. p. 102.

though errant and ignorant belief and intention. But judging "Spiritualism" from the writings of its acknowledged representatives, we are forced to the conclusion that in the main it is a repetition of the old story of imposture and credulity. "Things unknown," says Montaigne, "are the principal and true field of imposture, for as much as, in the first place, their very strangeness lends them credit; and, moreover, by not being subjected to our ordinary reason, they deprive us of the means to question and dispute them. On which account, says Plato, it is much more easy to satisfy the hearers, when speaking of the nature of the gods, than of the nature of men, because the ignorance of the auditory affords a fair and large career, and all manner of liberty in the handling of recondite things; and thence it comes to pass that nothing is so firmly believed as what we least know, nor any people so confident as those who entertain us with fables." (c. xxxi.)

---

